# *Azospirillum*: benefits that go far beyond biological nitrogen fixation

**DOI:** 10.1186/s13568-018-0608-1

**Published:** 2018-05-04

**Authors:** Josiane Fukami, Paula Cerezini, Mariangela Hungria

**Affiliations:** 1 Embrapa Soja, C.P. 231, Londrina, Paraná 86001-970 Brazil; 2 Department Biochemistry and Biotechnology, Universidade Estadual de Londrina, C.P. 60001, Londrina, Paraná 86051-990 Brazil

**Keywords:** Plant growth promoting bacteria, PGPB, Inoculant, Induced systemic resistance, Systemic acquired resistance, Induced systemic tolerance

## Abstract

The genus *Azospirillum* comprises plant-growth-promoting bacteria (PGPB), which have been broadly studied. The benefits to plants by inoculation with *Azospirillum* have been primarily attributed to its capacity to fix atmospheric nitrogen, but also to its capacity to synthesize phytohormones, in particular indole-3-acetic acid. Recently, an increasing number of studies has attributed an important role of *Azospirillum* in conferring to plants tolerance of abiotic and biotic stresses, which may be mediated by phytohormones acting as signaling molecules. Tolerance of biotic stresses is controlled by mechanisms of induced systemic resistance, mediated by increased levels of phytohormones in the jasmonic acid/ethylene pathway, independent of salicylic acid (SA), whereas in the systemic acquired resistance—a mechanism previously studied with phytopathogens—it is controlled by intermediate levels of SA. Both mechanisms are related to the NPR1 protein, acting as a co-activator in the induction of defense genes. *Azospirillum* can also promote plant growth by mechanisms of tolerance of abiotic stresses, named as induced systemic tolerance, mediated by antioxidants, osmotic adjustment, production of phytohormones, and defense strategies such as the expression of pathogenesis-related genes. The study of the mechanisms triggered by *Azospirillum* in plants can help in the search for more-sustainable agricultural practices and possibly reveal the use of PGPB as a major strategy to mitigate the effects of biotic and abiotic stresses on agricultural productivity.

## Introduction

Projections of population increases, especially in developing countries, as well as of life expectancy worldwide, imply greater needs for food and feed (FAO 2009). To achieve higher productivity, agriculture is being intensified, mainly with monocultures highly dependent on increased chemical inputs, including pesticides and fertilizers (McArthur and McCord 2017; Roser and Ritchie 2017). However, to ensure long-term food production, we must develop sustainable agricultural practices, based on conservationist practices, to achieve economic returns for farmers, but with stability in long-term production and minimal adverse impact on the environment (Sá et al. 2017). In this context, the use of microbial inoculants plays a key role, and we may say that we are starting a “microgreen revolution.”

The nomenclature “plant-growth-promoting bacteria (PGPBs)” has been increasingly used for bacteria able to promote plant growth by a variety of individual or combined mechanisms. By this definition, rhizobia—studied and used in commercial inoculants for more than a century—are also PGPBs. Undoubtedly, besides rhizobia, the most studied and used PGPB is *Azospirillum*, encompassing bacteria with a remarkable capacity to benefit a range of plant species (Bashan and de-Bashan 2010; Hungria et al. 2010; Hungria 2011; Fukami et al. 2016; Pereg et al. 2016).

The genus *Spirillum* was first reported by Beijerinck (1925), and decades later reclassified as *Azospirillum*, because of its ability to fix atmospheric nitrogen (N_2_), discovered and reported by the group of Dr. Johanna Döbereiner in Brazil, in the 1970s (Tarrand et al. 1978). After the discovery that *Azospirillum* was diazotrophic, several studies evaluated its capacity to fix N_2_ and to replace N-fertilizers when associated with grasses (Okon et al. 1983), including sugarcane (*Saccharum* spp.), grain crops such as maize (*Zea mays* L.), wheat (*Triticum aestivum* L.), and rice (*Oryza sativa* L.), pastures such as *Brachiaria* (= *Uruchloa*), among others (Lima et al. 1987; Cassán et al. 2015; Marks et al. 2015; Fukami et al. 2016; Hungria et al. 2016; Pereg et al. 2016). Twenty species of *Azospirillum* (DSMZ 2018) have been described so far, but *A. brasilense* and *A. lipoferum* have been the subjects of the highest numbers of physiological and genetic studies (Baldani and Baldani 2005; Fibach-Paldi et al. 2012).

Beneficial results have been obtained consistently with *Azospirillum* applied to a variety of crops (e.g. Okon and Labandera-Gonzalez 1994; Bashan et al. 2004; Pereg et al. 2016) in dozens of commercial inoculants worldwide (Okon et al. 2015). Intriguingly, although the Brazilian research group headed by Dr. Döbereiner contributed to dozens of studies with *Azospirillum* (Döbereiner and Pedrosa 1987; Reis et al. 2000; Baldani and Baldani 2005), it was only in 2009 that the first commercial inoculant containing *A. brasilense* started to be commercialized in the country (Hungria et al. 2010; Hungria 2011); however, more than 3 million doses of inoculants are now applied annually by farmers, for inoculation both of non-legumes and for co-inoculation of legumes.

**Fig. 1:**
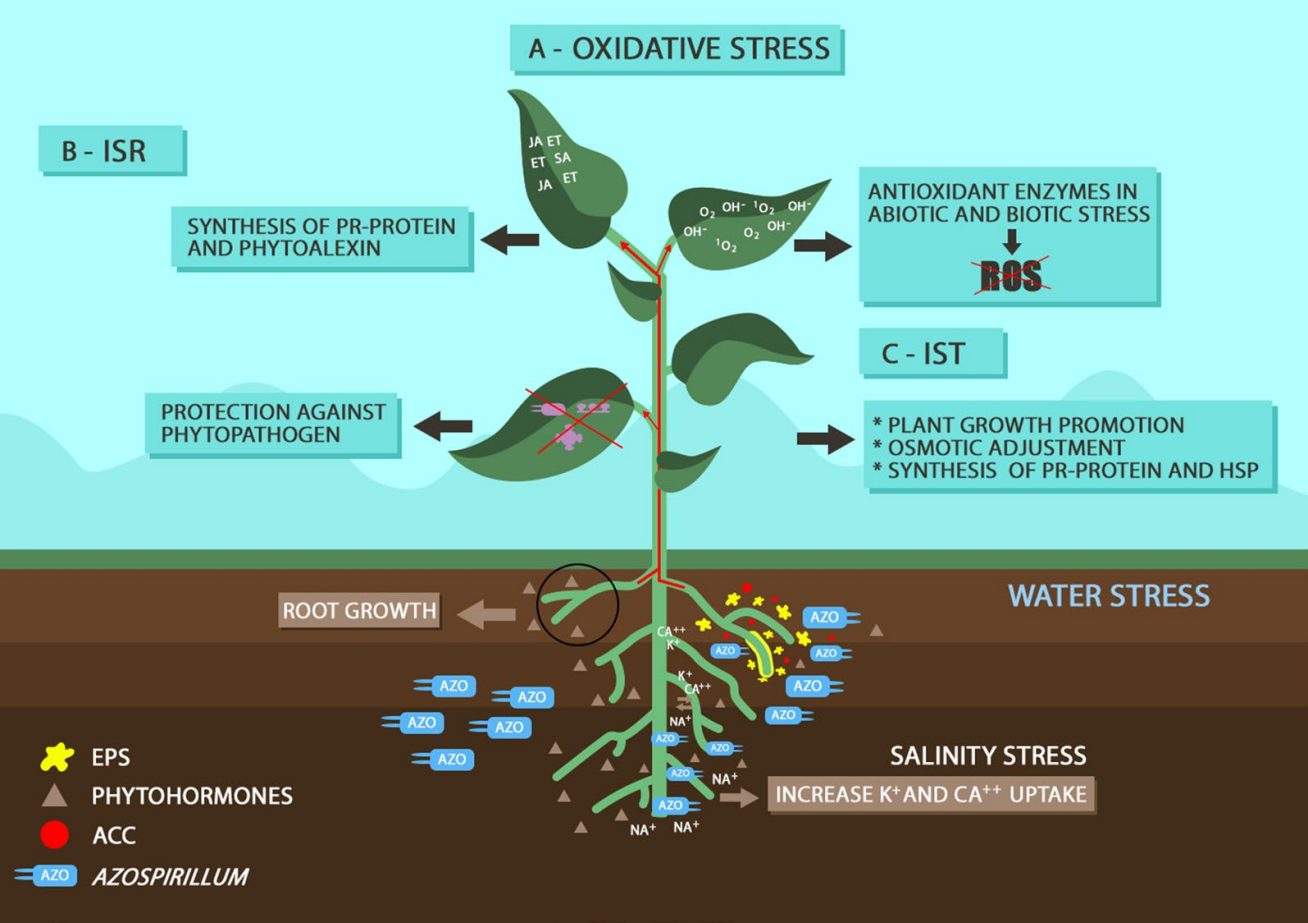
Mechanisms of tolerance of biotic and abiotic stresses induced by *Azospirillum* in plants. Tolerance to biotic stress include induced systemic resistance (ISR), mediated by increased levels of phytohormones in the jasmonic acid (JA)/ethylene (ET) pathway independent of salicylic acid (SA), and systemic acquired resistance (SAR)—a mechanism previously studied with phytopathogens—controlled by intermediate levels of SA. Tolerance of abiotic stresses, named as induced systemic tolerance (IST), is mediated by antioxidants, osmotic adjustment, production of phytohormones, and defense strategies such as the expression of pathogenesis-related (*PR*) genes

Although the most prevalent reported benefit of *Azospirillum* has been its capacity of fixing N_2_, an increasing number of studies describes other properties that imply growth-promotion. One main property of *Azospirillum* relies on the synthesis of phytohormones and other compounds, including auxins (Spaepen and Vanderleyden 2015), cytokinins (Tien et al. 1979), gibberellins (Bottini et al. 1989), abscisic acid (Cohen et al. 2008), ethylene (Strzelczyk et al. 1994), and salicylic acid (Sahoo et al. 2014). Phytohormones greatly affect root growth, resulting in improvements in uptake of moisture and nutrients (Ardakani and Mafakheri 2011). Some *Azospirillum* strains can solubilize inorganic phosphorus, making it more readily available to the plants and resulting in higher yields (Turan et al. 2012). There are also reports of *Azospirillum* helping in the mitigation of abiotic stresses, such as salinity and drought (Creus et al. 2004; Rodríguez-Salazar et al. 2009; Kim et al. 2012), by triggering induced systemic tolerance (IST) (Yang et al. 2009). *Azospirillum* has also been reported to help in the mitigation of excessive compost and heavy metals (Bacilio et al. 2003; de-Bashan et al. 2010). Another important feature of *Azospirillum* is related to biological control of plant pathogens (Bashan and de-Bashan 2002a, b; Khan et al. 2002; Romero et al. 2003; Tortora et al. 2011), enabled by the synthesis of siderophores, and limiting the availability of iron (Fe) to phytopathogens (Tortora et al. 2011), or causing alterations in the metabolism of the host plant, including the synthesis of a variety of secondary metabolites that increase plant resistance to infection by pathogens, a mechanism known as induction of systemic resistance (ISR) (Sudha and Ravishankar 2002; van Loon and Bakker 2005). Due to the several mechanisms reported to promote plant growth, Bashan and De-Bashan (2010) proposed the “theory of multiple mechanisms” in which the bacterium acts in a cumulative or sequential pattern of effects, resulting from mechanisms occurring simultaneously or consecutively. In this review we will give emphasis to the mechanisms of *Azospirillum* that can improve plant tolerance of biotic and abiotic stresses (Fig. 1).

## Oxidative stress

Abiotic and biotic stresses result in oxidative damage to plants due to an increase in reactive oxygen species (ROS), representing an initial mechanism of plant response to the attack of pathogens (Finkel 2000; León and Montesano 2013), and of defense against abiotic stresses (Heidari and Golpayegani 2012; Wang et al. 2012).

The ROS molecules encompass free radicals resulting from the oxygen (O_2_) metabolism, including superoxide radicals (O_2_^−^), hydroxyl radicals (OH^−^), hydrogen peroxide (H_2_O_2_), and singlet oxygen (^1^O_2_) (Bowler et al. 1992; Gill and Tuteja 2010). Under normal conditions, ROS are produced via the aerobic metabolism by the interaction between O_2_ and electrons escaping from the electron transport chain in the chloroplast and mitochondria (Halilwell and Gutteridge 1989). However, under stress conditions, ROS accumulation affects cellular components, causing damage to membranes by lipid peroxidation (Smirnoff 1993), and/or by the accumulation of solutes, such as proline and betaine, which may protect cells against increased levels of ROS (Chen and Murata 2002).

Oxidative stress is relieved in plants by antioxidant enzymes, such as superoxide dismutase (EC 1.15.1.1; SOD), catalase (EC 1.11.1.6; CAT), and ascorbate peroxidase (EC 1.11.1.11; APX) (Wisniewski-Dyé et al. 2012; Ozyigit et al. 2016). The enzyme SOD is the first in the defense against ROS, converting the radical superoxide (O_2_^−^) to H_2_O_2_, which is then removed by CAT and APX by the conversion of H_2_O_2_ to water (H_2_O) and (O_2_) (Lamb and Dixon 1997; Asada 1999). In general, ROS detoxification systems vary with plant species, genotypes, and age, as well as with the type and duration of stress (Hodges et al. 1996).

The genes that encode the detoxification enzymes are found in different compartments of plant cells, varying in number and location, depending on the plant species. SOD enzymes are divided into three groups, based on the cofactor metal: the copper/zinc (Cu/ZnSOD), iron (FeSOD), and manganese (MnSOD) classes (Jozefczak et al. 2015). The SOD system in maize consists of several isoenzymes; SOD2, SOD4, SOD4A, and SOD5 are found in the cytosol (Cu/ZnSOD), while SOD3 (MnSOD) is encoded by the *sod3* multigene family and located in the mitochondria (Jung et al. 2001).

APX isoenzymes in superior plants are encoded by a multigenic family (Ozyigit et al. 2016); the APX1 and APX2 cytosolic isoforms are the most important in the APX family in providing antioxidant protection (Shigeoka and Maruta 2014), induced mainly under extreme light conditions or heat stress (Davletova et al. 2005). In relation to the CAT isoenzymes, CAT1 and CAT2 are found in peroxisomes, glyoxysomes, and also in the cytosol (Scandalios et al. 1997), and CAT3 in the mitochondria (Jung et al. 2001).

Although early studies have focused on plant response to phytopathogens, there are indications that PGPBs may induce plant oxidative stress as an initial defense response, probably because plants perceive these microorganisms as potential threats. What is now known is that PGPB, including *Azospirillum*, are capable of inducing the synthesis of antioxidant enzymes in plants, reducing the deleterious effects of ROS (Han and Lee 2005; Heidari and Golpayegani 2012; Upadhyay et al. 2012; Fukami et al. 2017, 2018).

Although *Azospirillum* appears to lack host specificity in the promotion of plant growth (Pereg et al. 2016), there are also indications that strains may vary in determinants that will contribute to the adaptation to the rhizospheric niche, affecting plant-bacterial interactions (Wisniewski-Dyé et al. 2012). Therefore, mechanisms of oxidative stress may contribute to the success of plant colonization. For example, Drogue and collaborators (2014) reported that colonization of *A. lipoferum* strain 4B in the rice rhizosphere seems to involve genes related to the detoxification of ROS, and similar results were reported for *A. brasilense* strain Sp245 in *Arabidopsis thaliana* (Spaepen et al. 2014), wheat (Méndez-Gómez et al. 2015), and also for *A. brasilense* strains Ab-V5 and Ab-V6 in maize (Fukami et al. 2017, 2018).

## Plant defense mechanisms to biotic stresses

### Induced systemic resistance

Plants have several inducible mechanisms against pathogens attack. A classic example is the acquired systemic resistance (SAR), which is activated after infection by a necrotrophic pathogen, and confers resistance to plants against a broad spectrum of pathogens, as well as against secondary infections for weeks or months (Fu and Dong 2013).

Some PGPB also show the capacity of inducing plant defense mechanisms, conferring resistance to pathogenic bacteria, viruses, and fungi, termed ISR (induced systemic resistance) (Lugtenberg and Kamilova 2009). The ISR triggered by non-pathogenic microorganisms begins in the infected primary tissues and is systemically spread throughout the plant, increasing the defensive capacity of distant tissues against infection of pathogenic agents (van Loon and Bakker 2005; Dutta et al. 2008). Once induced, plants can remain protected for prolonged periods (van Loon 2007). This mechanism was first described by van Peer et al. (1991) in carnation (*Dianthus caryophyllus* L.), with protection against *Fusarium oxysporum* f. sp. *dianthi* via the synthesis and accumulation of phytoalexins resulting from inoculation with *Pseudomonas* sp. WCS417r. The mechanism was also described by Wei et al. (1991) in cucumber (*Cucumis sativus* L.), in which six out of the 94 strains of PGPB evaluated, encompassing five species of *Pseudomonas* and one of *Serratia*, protected the leaves against anthracnose caused by *Colletotrichum orbiculare.*

van Loon (2007) defined four main mechanisms by which PGPB may induce ISR in plants: (i) developmental, escape: related to plant-growth promotion; (ii) physiological, tolerance: reduction of symptom expression; (iii) environmental: associated with microbial antagonism in the rhizosphere; (iv) biochemical resistance: by induction of cell-wall reinforcement, of phytoalexins synthesis, of pathogenesis-related (PR) proteins, and “priming” of defense responses (resistance), enabling the plants to rapidly and effectively activate cellular defense responses that are induced by contact with the pathogen.

The ISR is also characterized by specific plant-PGPB interactions, implying that a PGPB that is capable of triggering ISR in a particular plant species may not be effective in another (van Loon 2007). The main group of PGPB that triggers ISR includes strains of the genera *Azospirillum*, *Pseudomonas* and *Bacillus* (Pérez-Montaño et al. 2014). A transcriptomic study of *Azospirillum* sp. strain B510 (isolated from cultivar Nipponbare) inoculated in rice induced one and repressed five *PR*-genes, whereas *A. lipoferum* strain 4B (isolated from cultivar Cigalon) induced more defense-related genes in Nipponbare than in Cigalon (Drogue et al. 2014). In another study with *A. thaliana*, *PR*-genes were induced when the plant was inoculated with *A. brasilense* strain Sp245 (Spaepen et al. 2014). *PR*-genes were also induced in maize inoculated with *A. brasilense* strains Ab-V5 and Ab-V6 (Fukami et al. 2017, 2018).

The SAR is associated with the synthesis and accumulation of salicylic acid (SA) in the plant, activating a coordinated expression of genes that encode PR-proteins (Kawagoe et al. 2015). One study demonstrated that NPR1 (“nonexpressor of *PR*-*gene1"*, related to the plant’s defense system) is an essential regulator in the SAR mechanism; it is transported to the cell nucleus in response to SA, where it acts as a transcriptional co-activator of a set of *PR*-genes (Pajerowska-Mukhtar et al. 2013; Pieterse et al. 2012, 2014), with an emphasis on *PR*-*1*, *PR*-*2*, and *PR*-*5* (Malamy et al. 1990; Uknes et al. 1992). The PR-proteins have different functions, some still unknown. We may cite as an example *PR*-*1* (a member of a multigene family) (Morris et al. 1998) with unknown biochemical function (van Loon et al. 2006), and *PR*-*2*, related to the synthesis of a β-1-3-glucanase (Kauffmann et al. 1987), which inhibits pathogenic fungal growth, since the main structural components of the cell wall of these microorganisms are chitin and β-glucan.

The activation/repression of *PR*-genes, mediated by NPR1, is tightly related to the levels of SA in plants. NPR1 assists in the activation of programmed cell death, acting as a negative regulator (Caarls et al. 2015). When the levels of SA are low, NPR4 (a paralog of NPR1) interacts with NPR1, leading to its degradation. Likewise, when the levels of SA are high, the binding between NPR1 and NPR3 (a paralog of NPR1) is promoted, and also results in the removal of NPR1 (Fu et al. 2012). When the SA level is intermediate, the interaction between NPR1 and NPR3 is suppressed, leading to the accumulation of NPR1, and activating the SA-dependent defense genes (Caarls et al. 2015).

In the case of ISR, studies on different species of PGPB and plants have established that the nature of the induced resistance, in most cases, is independent of SA (Yan et al. 2002; De Vleesschauwer et al. 2008; Segarra et al. 2009) and, in general, is associated with signaling molecules, such as jasmonic acid (JA) and its derivatives (such as jasmonate), and ethylene (ET) (Glick 2012; Ahemad and Kibret 2014), involving the induction of PR-proteins, such as PR-3 and PR-4 (chitinase family), and PDF1.2 (a plant defensin) (van Loon and van Strien 1999; Gond et al. 2015). In a study with strawberry (*Fragaria ananassa*) inoculated with *A. brasilense* REC3, Elias et al. (2018) reported increased ET synthesis and up-regulation of genes associated with ET signaling (Faetr1, Faers1, Faein4, Factr1, Faein2 and Faaco1), supporting the hypothesis of priming activation characteristic of ISR mediated by this PGPB.

There is evidence that the mechanisms of ISR, with signaling by JA/ET, are different from SAR, mediated by NPR1 (Spoel 2003; Stein et al. 2008; Pieterse et al. 2012; Pieterse and Van Wees 2015). The evidence corroborates the results of Yasuda et al. (2009), in which rice plants inoculated with *Azospirillum* sp. B510 increased the plant resistance to the pathogenic fungus *Magnoporthe oryzae* and to the bacterium *Xanthomonas oryzae*, through mechanisms independent of SA-signaling, with no accumulation of SA or PR-proteins. Similar results were described by De Vleesschauwer and collaborators (2008) for *P. fluorescens* WCS374r. However, other studies using cells and metabolites of *A. brasilense* Ab-V5 and Ab-V6 applied by different methods resulted in the induction of *PR*-*1* SAR-related and *PRP*-*4* ISR-related genes (Fukami et al. 2017).

Several studies have demonstrated that the exogenous applications of SA (Bari and Jones 2009) and JA (Lorenzo and Solano 2005; Wasternack 2007; Bari and Jones 2009) in plants induce *PR*-genes, resulting in increased resistance to various phytopathogens. Agrawal and collaborators (2000) reported the first evidence of exogenous application of JA as an effective inducer of the *PR1* family in rice. There are also reports of the application of ISR-inducing chemicals, such as JA or SA, in reducing the incidence of diseases in rice. However, the application of purified exopolysaccharides (EPS) of *Azospirillum* also conferred resistance against the fungus *Pyricularia oryzae* (Sankari et al. 2011), suggesting that EPS may represent another alternative for increasing ISR.

## Plant defense mechanisms to abiotic stresses

Plants are commonly exposed to several environmental stresses such as high and low temperatures, drought, salinity, alkalinity, UV-rays (Sharma et al. 2012); estimates are that about 30% of the global crop production is lost as a result of abiotic stresses (Goswami et al. 2016), and PGPB can play a strategic role in reducing these losses, by activating several physiological and biochemical tolerance mechanisms in plants (Yang et al. 2009; Kim et al. 2012; Sarma et al. 2012), named induced systemic tolerance (IST). The mechanisms related to IST include antioxidant defense (Heidari and Golpayegani 2012; Wang et al. 2012), osmotic adjustment (Sarma and Saikia 2014), production of phytohormones such as indole-3-acetic-acid (IAA) (Spaepen and Vanderleyden 2015), defense strategies such as the expression of *PR*-genes (Kim et al. 2014), and the induction of heat-shock proteins (HSP) (Lim and Kim 2013).

### Saline stress

Salinity is considered one of the most critical abiotic stresses, impacting agricultural productivity and sustainability due to reductions in photosynthesis, respiration, and protein synthesis (Ahmad and Prasad 2012; Dwivedi et al. 2015). Salinity also causes nutritional disturbances in plants that lead to the deficiency of various nutrients and the increase in sodium (Na^+^) levels (Zahedi et al. 2012). First, the high concentration of salt in the rhizosphere affects water absorption by the plants; subsequently, toxic ionic concentrations inside the plants result in inhibition of many physiological and biochemical processes, such as the absorption and assimilation of nutrients (Hasegawa et al. 2000; Munns and Tester 2008).

Plants use many important adaptive mechanisms to deal with the adverse effects of salinity, one of them being the accumulation of solutes, including amino acids (proline), sugars (mannitol), and quaternary ammonium (glycine betaine), which help to maintain the water within the cells, combating dehydration (Nuccio et al. 1999). Another mechanism is the increase in ROS synthesis in cells (Gururani et al. 2013), as well as of the cytosolic expression of APX (Torsethaugen et al. 1997).

Among the PGPB, the genus *Azospirillum*—with an emphasis on *A. brasilense*—is probably the most studied microorganism for the mitigation of salinity stress in various cultures (Creus et al. 2004; Barassi et al. 2006; Rodríguez-Salazar et al. 2009; Carrozzi et al. 2012; Fasciglione et al. 2015). Example of effects of *Azospirillum* include the study by Hamdia and collaborators (2004), that reported that the inoculation of *Azospirillum* spp. in cultivars of maize altered the selectivity of Na^+^, K^+^, and Ca^++^ ions, by restricting Na^+^ absorption and increasing K^+^ and Ca^++^ uptake; the protective role of the bacterium was verified by the reduction in proline content, and also by plant-growth promotion. Likewise, plant-growth promotion and lower accumulation of solutes were also reported by Fukami et al. (2017) in maize inoculated with *A. brasilense* Ab-V6, but not with Ab-V5, indicating differences between strains. In addition, Fukami et al. (2017) observed that inoculation with strain Ab-V6 induced the expression of genes related to antioxidant enzymes, and similar results were reported when different species of *Azospirillum* were used in inoculants applied to canola (*Brassica napus* L.) (Baniaghil et al. 2013).

In another study, inoculation with *A. brasilense* strain NH, but not with Sp7 (Nabti et al. 2009), was very effective in restoring the vegetative growth and seed yield of durum wheat (*Triticum durum* var. Waha) grown with 160 and 200 mM NaCl, reducing the accumulation of proline and total sugars (Alamri and Mostafa 2009). Other studies reported beneficial effects of inoculation of *A. brasilense* on sweet pepper (*Capsicum annuum* L.) (Amor and Cuadra-Crespo 2012), and white clover (*Trifolium repens*) (Khalid et al. 2017).

It is worth mentioning that several rhizobial strains can also help to increase plant tolerance of salinity, as has been reported for pea (*Pisum sativum* L.), fava beans (*Vicia faba* L.) (del Cordovilla et al. 1999), common bean (*Phaseolus vulgaris* L.) (Dardanelli et al. 2008; Fukami et al. 2018), and also in non-legumes as lettuce (*Lactuca sativa* L.) (Han and Lee 2005). This may be due, at least partially, to the ability of some rhizobial strains to synthesize phytohormones (Yanni and Dazzo 2015; Imada et al. 2017), increasing root growth, a property that is expanding their use as PGPB also in non-legumes (Askary et al. 2009; García-Fraile et al. 2012; Hasan et al. 2014; Yanni and Dazzo 2015).

### Drought stress

Drought is another major limitation to crop production worldwide (Lesk et al. 2016), and global climate changes are increasing the frequency of negative reports. Many mathematical models predict reductions in rainfall and increases in temperatures by 2050 (IPCC 2014; Shanker et al. 2014), resulting in agricultural losses for economically important crops, and impacting food security (Foley et al. 2011; IPCC 2014). Thus, there is need to increase drought tolerance in crops and increase yields under conditions of depleted moisture availability (Ngumbi and Kloepper 2016).

Moisture shortage in plants affects stomatal function, which reduces the leaf CO_2_/O_2_ ratio, inhibiting photosynthesis with concomitant reduction of biomass production (Gilbert et al. 2011; Lopes et al. 2011; Mutava et al. 2015). Under severe conditions, drought induces oxidative stress in plants, resulting from the accumulation of ROS (Souza et al. 2013; Silva et al. 2014). The plant responds with the synthesis and activity of several antioxidant enzymes, such as CAT, peroxides (POX), SOD, glutathione peroxidase (GPX), and APX (Simova-Stoilova et al. 2008). In addition, other strategies such as osmotic adjustment, maintenance of root viability, membrane stability, and accumulation of proteins and other metabolites—including proline, glycine betaine, and trehalose—help, directly or indirectly, in the maintenance of plant metabolism under drought stress (Huang et al. 2014; Cohen et al. 2015; Ngumbi and Kloepper 2016).

Inoculation with PGPB may be strategic to increase drought tolerance (Marulanda et al. 2007), since these microorganisms can elicit IST (Yang et al. 2009). In addition, PGPB may help plant-drought tolerance by the production of EPS (Sandhya et al. 2010), phytohormones (Dodd et al. 2010; Fibach-Paldi et al. 2012), 1-aminocyclopropane-1-carboxylate (ACC) deaminase (Lim and Kim 2013), volatile compounds, inducing the accumulation of osmolytes (Cohen et al. 2015), antioxidants, up- or down-regulation of stress-responsive genes (Ngumbi and Kloepper 2016; Vurukonda et al. 2016), and changes in root morphology (Rodríguez-Salazar et al. 2009; Cohen et al. 2015).

In a pioneer study of the effects of PGPB in plant-gene expression, Timmusk and Wagner (1999) reported that the inoculation of *A. thaliana* with *Paenibacillus polymyxa* induced the drought-responsive gene *ERD15* (early response to dehydration). In another study, inoculation with *Pseudomonas* spp. compensated the drought effects with an enhanced synthesis of proline, amino acids, and soluble sugars, which resulted in better absorption of moisture and nutrients and enhanced plant growth (Sandhya et al. 2010). Furthermore, *Pseudomonas* strains produced abundant EPS under stress, providing a micro-environment that favored water maintenance, and protected both the microorganism and the plant against dehydration (Alami et al. 2000; Sandhya et al. 2010).

In various studies, the role of *Azospirillum* in mediating drought tolerance has been documented (Bano et al. 2013; Cohen et al. 2015; Hungria et al. 2015; Saeed et al. 2016; Curá et al. 2017). Noteworthy, drought tolerance of *Azospirillum* was reported even in drastic conditions of deserts (Bashan et al. 2012). Positive effects have been attributed to the synthesis of abscisic acid (ABA), inducing stomatal closure (Cohen et al. 2015), as well as to the accumulation of solutes such as free amino acids and soluble sugars, which help mitigate dehydration. *Azospirillum* also improves plant traits that can help tolerance of water deficit, such as root branching, increased root biomass, increased density of root hairs (Cassán and García de Salamone 2008; Lopes et al. 2011; Hungria et al. 2015), which foster exploration of the water in the soil; improvements in plant-root activity have been explained in terms of the action of phytohormones synthesized by PGPB, such as IAA (Saharan and Nehra 2011).

In a study performed by Saeed and collaborators (2016), when canola seeds were inoculated with *A. lipoferum,* there were increases in percentage germination, in root-surface area and in chlorophyll content, and improvement in water potential under drought conditions. In another study, *A. brasilense* increased *Arabidopsis* growth, proline levels, photosynthetic and photoprotective pigments, and decreased stomatal conductance and water losses under drought, attributes that were correlated with increases in ABA levels (Cohen et al. 2015). More recently, Curá et al. (2017) demonstrated that inoculation of maize with *A. brasilense* or *Herbaspirillum seropedicae* improved plant tolerance to desiccation, effects correlated with ABA and ethylene contents. Therefore, the use of PGPB strains—especially *Azospirillum*—is promising for the mitigation of drought effects on crop plants. However, it is important to consider that strains of *Azospirillum* may differ in their properties the confer tolerance of drought, justifying a selection of the most effective ones (García et al. 2017).

Remarkably, inoculation with *Azospirillum*, a typical rhizospheric bacterium, via foliar spray can also increase plant growth (Fukami et al. 2016), attributable to the synthesis of IAA by the bacterium, i.e. a plant-signaling process mediated by the bacterium, far stronger than when synthetic IAA was applied (Puente et al. 2017). Intriguingly, in maize, foliar application of *Azospirillum* also elicited genes related to tolerance of abiotic stresses (*APX1*, *APX2*, *SOD4*), as well as defense genes *(PR*-genes), which has also been attributed to phytohormones signaling (Fukami et al. 2017). Also in brachiaria (*U. ruziziensis*), foliar application of *Azospirillum* Ab-V5 and Ab-V6 increased the tolerance of water stress, by increasing the activity of enzymes related to the removal of reactive oxygen species, protecting chlorophyll a (Bulegon et al. 2016).

We may also consider that different microorganisms and microbial processes can be combined to make agriculture more sustainable and productive, helping to mitigate the impacts of abiotic stresses. One important example relies on the co-inoculation of rhizobial and non-rhizobial PGPB, with several reports of increased yields, for example, with soybean (Hungria et al. 2013; Pérez-Montaño et al. 2014; Chibeba et al. 2015; Cerezini et al. 2016; Puente et al. 2017), and common bean (*Phaseolus vulgaris* L.) (Hungria et al. 2013). In the co-inoculation with rhizobia, *Azospirillum* usually contributes with root-growth promotion (Cassán et al. 2009; Juge et al. 2012), allowing precocity and increased nodulation by the rhizobia (Chibeba et al. 2015). Most importantly, Cerezini et al. (2016) have shown that soybean co-inoculation with *Azospirillum* and *Bradyrhizobium* increased grain yield under moderate water restriction (Cerezini et al. 2016), representing a promising technology for the mitigation of abiotic stresses.

## Final remarks

*Azospirillum* is currently one of the most broadly studied and commercially employed PGPB. Previous studies with *Azospirillum* emphasize its capacity of fixing atmospheric N_2_, followed by benefits in promoting plant growth via synthesis of phytohormones. More recently, it has been shown that the benefits should be extended to the capacity of some *Azospirillum* strains to protect plants from biotic stresses, triggering ISR defense mechanisms, and from abiotic stresses, through IST. Figure 1 summarizes the mechanisms discussed in this review of tolerance of abiotic and biotic stresses promoted by inoculation of *Azospirillum* in plants, encompassing detoxification of oxidative stress, ISR and IST. The mechanisms that PGPB use to cope with biotic and abiotic stresses vary with the plant species and cultivar and with the bacterial species and strains, and also depend on the phytopathogen and the intensity of the abiotic stress. Further studies to elucidate the mechanisms of action of PGPB—as well as of the response of plants to stresses—are of fundamental importance for understanding the potential and increasing the use of PGPB as an important and sustainable strategy to mitigate the effects of biotic and abiotic stresses in agriculture.
